# Perceptions, Attitudes, and Use of AI by Medical Students: Mixed Methods Study

**DOI:** 10.2196/91345

**Published:** 2026-07-20

**Authors:** Frédéric Paris, Vincent Garrouste, Laure Abensur Vuillaume

**Affiliations:** 1 University Hospital of Geneva, Rue Gabrielle-Perret-Gentil 4, Geneva, 1205, Switzerland, 41 22 372 33 11; 2 Centre hospitalier universitaire d'Orléans, Orléans, Centre-Val de Loire, France; 3 Centre Hospitalier Régional de Metz-Thionville, Metz-Thionville, Grand Est, France

**Keywords:** artificial intelligence, AI, medical education, attitude, knowledge, students, curriculum design

## Abstract

**Background:**

AI is transforming medicine by enhancing care, reducing administrative tasks, and facilitating research. AI also raises many concerns, including a lack of clinical context awareness, data dependence, and the absence of ethical judgment. As future practitioners, medical students must be prepared for these changes. Most studies assessing students’ attitudes and knowledge were conducted before AI became accessible and tailored to the needs of the population. Therefore, how medical students actually use AI remains largely unexplored.

**Objective:**

This study aimed to explore French medical students’ perceptions, attitudes, and use of AI.

**Methods:**

A mixed methods study was conducted in 2025 among French medical students in their clerkship year. An online survey included open-ended questions about the definition of AI and feelings toward AI, a Likert scale item to assess specific attitudes, and multiple-choice questions about student characteristics. Quantitative analysis was performed using Kruskal-Wallis tests, chi-square tests, and exploratory multivariable linear regression to examine associations between AI knowledge, attitudes, and student characteristics. Qualitative thematic analysis was conducted inductively on open-text responses regarding perceptions of AI, feelings, training expectations, and use.

**Results:**

Of the 1377 responses received, 1342 were included. Students had a median age of 23 (IQR 22-24) years and were predominantly in their fifth year. Only 5% (67/1342) provided a correct definition of AI, while 57.4% (770/1342) gave incorrect responses. Attitudes toward AI were generally positive, with a median score of 7 (IQR 5-8). Students with unknown AI definitions had significantly lower attitude scores (*P*=.02), although the magnitude of the difference was small (ε²=0.005). In multivariable analysis, belonging to the unknown AI definition category remained associated with a lower general attitude toward AI score compared with the incorrect category (regression coefficient β=−0.72, 95% CI −1.17 to −0.28; *P*=.001). Regarding education, 48.5% (651/1342) of students preferred AI training outside the formal curriculum. Qualitative analysis revealed 5 themes: representation, nuanced optimism, critical consideration, replacement, and AI use. Students describe AI as a robot, an improved search engine, or an unlimited data source. Their nuanced optimism blends enthusiasm for efficient patient care and the opportunity to focus more on the patient relationship, with concerns about dehumanization, energy costs, and skill regression. Critical consideration underscores distrust from ethical dilemmas and data security risks. Replacement concerns arise from shifting professional roles, though many believe human empathy remains irreplaceable. Regarding AI use, students highlight its potential for administrative aid, personalized training, and clinical support.

**Conclusions:**

Medical students report generally positive but varied attitudes toward AI, despite having a limited understanding of its foundations. Ecological concerns, fears of skill loss, and ethical issues coexist with widespread self-directed use of AI for learning. These findings provide a descriptive baseline for future evaluations of AI-related training, particularly regarding critical appraisal, ethical issues, and self-directed AI use.

## Introduction

AI traditionally refers to "any machine or algorithm capable of observing its environment, learning, and, through its experiences and acquired knowledge, performing intelligent actions or making decisions” [1]. It is recognized as potentially having a significant impact on care delivery and clinical practices [2]. Indeed, AI, particularly through deep learning applications, has shown significant potential to improve diagnosis, reduce administrative tasks, and facilitate research [3]. However, AI appears to have limitations in this field, particularly a lack of contextual understanding, data dependence, an absence of ethical judgment, and difficulty innovating beyond existing knowledge [4]. Historically, the first medical applications of AI were studied in radiology, infectious diseases, and ophthalmology [5]. Since 2020, the scope of AI applications has been constantly expanding across various medical specialties, such as reducing administrative burden in emergency departments through AI systems like “KATE,” developed to reduce triage delays, and “SCLEPIOS,” which can generate consultation reports and perform coding in emergency settings [6]. This phenomenon is also observed in health education across various fields, including clinical supervision, recruitment, clinical reasoning, intelligent tutoring systems, and evaluation [7]. These innovations, coupled with the rise of educational chatbots, illustrate the growing integration of intelligent tools in clinical training and practice [8].

Medical students are directly impacted by the use of AI in clinical settings. Moreover, several concerns related to the use of AI are progressively emerging [9-11]. These concerns include the potential decline of certain skills, the lack of acquisition of specific competencies, and an increasing dependence on digital tools [9,10]. Such dependence, along with the risk of skill degradation, may affect students’ abilities in synthesis and critical thinking. However, these risks remain poorly documented in clinical contexts to date [9-12].

Therefore, understanding their use, attitudes, and comprehension of AI during their studies seems essential to adapt educational programs and prepare future physicians to work effectively with these new technologies [13,14]. Early studies exploring medical students’ attitudes focused on radiology [15]. Authors showed a decreased interest in radiology as a specialty among students due to fear of being replaced by AI [16-18]. Subsequently, several studies also explored medical students’ knowledge and attitudes about AI [7,19,20]. It was shown that students had limited knowledge of AI [21,22], with media being their main source of learning [23]. Regarding their attitudes, students recognized AI’s usefulness for diagnosis, research, and administrative task assistance. However, they expressed concerns about ethical issues, the weakening of doctor-patient relationships, and AI’s limitations in unexpected scenarios [24-27]. Finally, one of the main themes among students was anxiety about being replaced by AI [24]. These attitudes may be influenced by students’ level of education and prior knowledge [28]. Moreover, students’ intention to use AI appears to be primarily influenced by social factors rather than AI’s intrinsic performance [29].

However, we found no studies evaluating French medical students’ knowledge and attitudes about AI. Additionally, most studies were conducted several years ago, when AI use was less widespread, and there were few concrete clinical applications. The attitudes and knowledge of AI, influenced by the recent and increasing use of AI by medical students, remain largely understudied today [30,31]. Finally, the use of AI among medical students remains underexplored [32]. Therefore, the knowledge and attitudes of students who have used AI since high school need to be explored [33]. The aim of this study was to explore French medical students’ perceptions, attitudes, and use of AI in 2025.

## Methods

We conducted a mixed methods qualitative and quantitative study using a survey that consisted of multiple-choice questions, a Likert scale, and short open-ended questions (SAQs). This study used an existing, validated survey originally developed by Teng et al [21].

### Population

To be included, participants had to be medical students aged 18 years or older, enrolled in a French medical school, and in their fourth to sixth academic years of study. These students are in the second cycle of medical studies, which corresponds to the clerkship year. Their training is divided between university teaching and clinical rotations in university hospitals, peripheral hospitals, and general practice offices.

Exclusion criteria included refusal to participate, incomplete questionnaire responses, and duplicate email addresses to prevent redundancy.

### Recruitment

The recruitment process involved several steps to obtain a representative sample of medical students in France.

First, an email containing an informational letter was sent to all medical schools in France. Then, dissemination was conducted through the social networks of the student association.

With approximately 25,000 medical students in 35 faculties hosting clinical students, our goal was to achieve a 10% participation rate, or 2500 students.

To motivate students to respond to the questionnaire, we offered students who completed the questionnaire the opportunity to participate in a contest to win one of five €20 (US $22.86) Amazon or Fnac gift cards.

### Survey

We used a published survey to assess the perceptions and attitudes of medical students regarding AI [21].

The questionnaire consisted of 3 sections (Multimedia Appendix 1): the first section asked about the participants' personal characteristics. The second section was an SAQ where participants were asked to describe what AI is. Finally, the last section consisted of several questions with Likert scale responses ranging from 1 to 10 about their attitude toward AI, as well as an SAQ about their feelings toward AI.

The original survey was designed for all Canadian health students. We moved the first section to the last to better meet the needs and interests of medical students (Multimedia Appendix 1). To adapt the questionnaire to our target population, we modified the institution question to ask about their medical school in France, their academic year, their self-assessment of their class ranking, and their career aspirations.

The survey was tested by 6 medical students to verify comprehension, identify errors, and assess completion time. The medical students, organized into 3 pairs, were informed of the study’s objectives and were encouraged to verbalize any confusion, identify errors, or request clarifications at any point during the process. During the pilot test, the first pair of participants highlighted 2 issues: a duplicated question, which was subsequently removed, and a multiple-choice question that was initially designed to allow only 1 response despite requiring multiple selections, which was corrected to permit multiple answers. After these revisions were made, the remaining 2 pairs completed the survey without encountering any further errors or comprehension difficulties. Importantly, none of the 6 participants reported any confusion or requested additional explanations, indicating that the questions were clear and well understood. The average time required to complete the survey was observed to be 5 minutes, confirming that the questionnaire was both concise and comprehensive for the intended audience.

### Data Analysis

Continuous and score variables were described using medians and IQRs, whereas categorical variables were summarized as counts and percentages. Questions allowing multiple responses were analyzed as separate binary variables for each response option; therefore, percentages may exceed 100%.

The primary grouping variable was the final AI definition category, obtained after adjudicating the free-text definitions according to the Organisation for Economic Co-operation and Development (OECD)–based coding framework: An AI system is a machine-based system that, for explicit or implicit objectives, infers from the input it receives how to generate outputs such as predictions, content, recommendations, or decisions that can influence physical or virtual environments. Different AI systems vary in their levels of autonomy and adaptiveness after deployment [34]. Interrater reliability between the 2 independent coders was assessed using raw agreement and weighted Cohen κ. Because AI definition categories were ordinal (unknown, incorrect, partially correct, and correct), weighted κ coefficients were calculated using both linear and quadratic weights. Bootstrap resampling was used to estimate 95% CIs for weighted κ.

Comparisons across final AI definition categories were performed using the Kruskal-Wallis test for continuous or score variables and the chi-square test for categorical variables. Effect sizes were reported for all comparisons: epsilon-squared for Kruskal-Wallis tests and Cramér *V* for chi-square tests. For variables with a significant Kruskal-Wallis test, pairwise post hoc Dunn tests were performed with Holm correction for multiple comparisons.

The general attitude toward AI score (0‐10) was analyzed as the main quantitative outcome. Six additional AI attitude items were analyzed separately as secondary outcomes. To limit multiplicity, *P* values from the 6 omnibus Kruskal-Wallis tests were adjusted using the Holm method. Post hoc Dunn-Holm comparisons were only interpreted for outcomes with a significant omnibus result after correction.

As an exploratory multivariable analysis, a linear regression model with heteroskedasticity-consistent robust SEs was fitted using the general attitude toward AI score as the dependent variable. Covariates included the final AI definition category, age, academic year, class ranking, and career aspirations (hospital-based clinical work, research, and private practice, each coded as yes or no). Results are reported as β coefficients with 95% CIs. All tests were 2-sided, and *P* values of less than .05 were considered statistically significant.

The qualitative component focused on free-text responses collected through the open-ended items of the survey. For the question assessing students’ definitions of AI, only the incorrect responses were analyzed, as the intention was to explore students’ misconceptions and the underlying representations associated with them. Additional free-text responses regarding feelings toward AI and expectations for AI training were also included in the qualitative dataset.

All qualitative data were compiled in Microsoft Excel and analyzed using a thematic analysis approach. Coding was performed iteratively: an initial set of codes was developed deductively based on concepts previously described in the literature (eg, nuanced optimism and replacement concerns), and additional categories were informed inductively from the data. Codes were progressively merged into broader categories through constant comparison, resulting in the identification of overarching themes. To enhance the reliability of the analysis, a second reviewer independently coded 30% of the material, and discrepancies were resolved through discussion.

Given the large and fully anonymous dataset, data saturation was not assessed, and validation by participants could not be performed. Representative quotations were selected to illustrate each theme. The study adhered to the COREQ (Consolidated Criteria for Reporting Qualitative Research) guidelines (Checklist 1).

All data were compiled in Excel version 2019 (Microsoft), and descriptive and qualitative analyses were performed using Microsoft Excel version 2019. Statistical tests were conducted using R software (version 4.3.1; R Foundation for Statistical Computing).

### Ethical Considerations

All authors confirm that all methods were applied in accordance with current guidelines and regulations. This study did not require specific authorization under current French regulations, as it is research evaluating the value of teaching through a student health survey. This research does not fall under clinical research and, in France, does not require ethics committee approval (French Public Health Code: articles L1110-1 to L6441-1) [35].

To ensure full compliance with ethical standards, we implemented rigorous safeguards throughout the study. No sensitive personal data, such as health information, ethnic origin, or any identifiable details, were collected at any stage. Participant anonymity was strictly preserved: Responses were untraceable, with no collection of IP addresses or other identifying metadata, and all data were analyzed in aggregated form to prevent individual recognition. Informed consent was secured through a comprehensive information letter provided to all participants prior to the survey, clearly outlining the study’s purpose, the voluntary nature of participation, and the intended use of data, with completion of the survey serving as implied consent. A request for consent was clearly written. Finally, data storage and security adhered to the General Data Protection Regulation (GDPR) requirements, with all responses encrypted, accessible only to the research team, and permanently deleted following analysis in 3 years. The email addresses were deleted immediately after the study.

## Results

### Statistical Analyses

#### Population Characteristics

**Figure 1.**
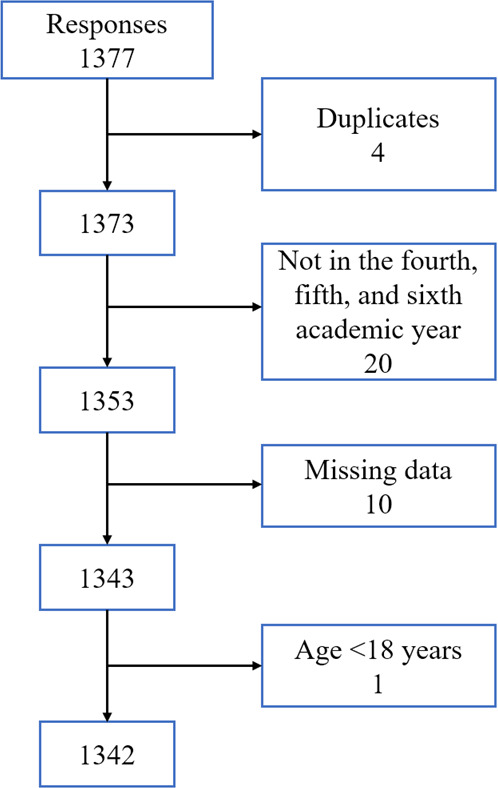
Study flowchart of participant inclusion.

The survey was available online from May 8, 2025, to June 16, 2025. Our study collected 1377 responses, of which 1342 met the inclusion criteria and were included in the final analysis (Figure 1) from 23 faculties. The demographic characteristics of the sample were as follows: a median age of 23.1 (IQR 22‐24) years, and the majority of students were in their fifth academic year (n=674, 50.2%; Table 1). Most participants selected hospital-based clinical work as a career aspiration (n=775, 57.7%) and were in the middle third of their class (n=694, 51.7%). The median general attitude toward AI score was 7 (IQR 5-8) [5-8]. Most students expected AI to affect their future career within less than 5 years (n=570, 42.5%). Concerning AI education, 651 (48.5%) preferred AI learning outside the formal curriculum, whereas 586 (43.7%) preferred its inclusion within the curriculum. The three most frequently selected educational objectives were identifying ways in which AI could improve healthcare quality (n=949, 70.7%), identifying when AI is appropriate in a given clinical context (n=916, 68.3%), and identifying the ethical implications of AI use in clinical settings (n=897, 66.8%). The geographic distribution showed an overrepresentation of students from Lille, Paris, and Nancy.

Figure 1 illustrates the flowchart showing the number of inclusions and exclusions, demonstrating how, out of the 1377 questionnaires received, we included 1342.

Table 1 and Textbox 1 show that the majority of students are in their fifth year, a minority wishes to pursue a research career, and a minority considers themselves to be in the lower third of their class ranking.

Textbox 1 shows that the 3 cities with the most responses are Lille, Paris Sorbonne, and Nancy.

**Table 1.** Characteristics and perceptions of the study population^a^.

Variable	Total study population (N=1342)
Age (y), median (IQR)	23 (22-24)
Academic year, n (%)	
Fourth	423 (31.5)
Fifth	674 (50.2)
Sixth	245 (18.3)
Career aspiration, n (%)	
Hospital	775 (57.7)
Research	382 (28.5)
Private practitioner	502 (37.4)
Class ranking, n (%)	
Lower third	190 (14.2)
Middle third	694 (51.7)
Upper third	458 (34.1)
Final AI definition category, n (%)	
Unknown	66 (4.9)
Incorrect	770 (57.4)
Partially correct	439 (32.7)
Correct	67 (5.0)
General attitude toward AI (0‐10)	7 [5-8]
Perceived timeline before AI will impact career, n (%)	
Less than 5 years	570 (42.5)
In 5 years	357 (26.6)
In 10 years	349 (26)
In 20 years	56 (4.2)
In 50 years	4 (0.3)
Not during my lifetime	6 (0.4)
Preferred place for AI learning, n (%)	
In curriculum	586 (43.7)
Outside curriculum	651 (48.5)
Other or free text	105 (7.8)
Top 3 educational objectives, n (%)	
Identify ways in which AI can improve health care quality	949 (70.7)
Identify when the technology is appropriate for a given clinical context	916 (68.3)
Identify the ethical implications of AI use in clinical settings	897 (66.8)

a Values are presented as median [IQR] or n (%). For career aspirations and educational objectives, multiple responses were allowed; therefore, percentages may exceed 100%.

Textbox 1. **Participation of French medical faculties.**

**With ≥100 respondents (%)**

- Lille 299 (22%)
- Paris Sorbonne 160 (12%)
- Nancy 109 (8%)

**With 50‐99 respondents (%)**

- Caen 98 (7%)
- Paris Cité 98 (7%)
- Strasbourg 75 (6%)
- Besançon 65 (5%)
- Marseille 59 (5%)
- Toulouse 57 (4%)
- Rennes 51 (4%)
- Limoges 50 (4%)

**With <50 respondents (%)**

- Amiens 42 (3%)
- Lyon 37 (3%)
- Paris Bicêtre 30 (2%)
- Tours 27 (2%)
- Antilles 26 (2%)
- Grenoble 21 (2%)
- Montpellier 20 (1%)
- Paris d’Orsay 13 (1%)
- Nîmes 2 (<1%)
- Nantes 1 (<1%)
- Reims 1 (<1%)
- Rouen 1 (<1%)

#### Definition of AI

After the adjudication of AI definitions, 770 (57.4%) definitions were categorized as incorrect, 439 (32.7%) as partially correct, 67 (5.0%) as correct, and 66 (4.9%) as unknown (Table 1).

**Figure 2.**
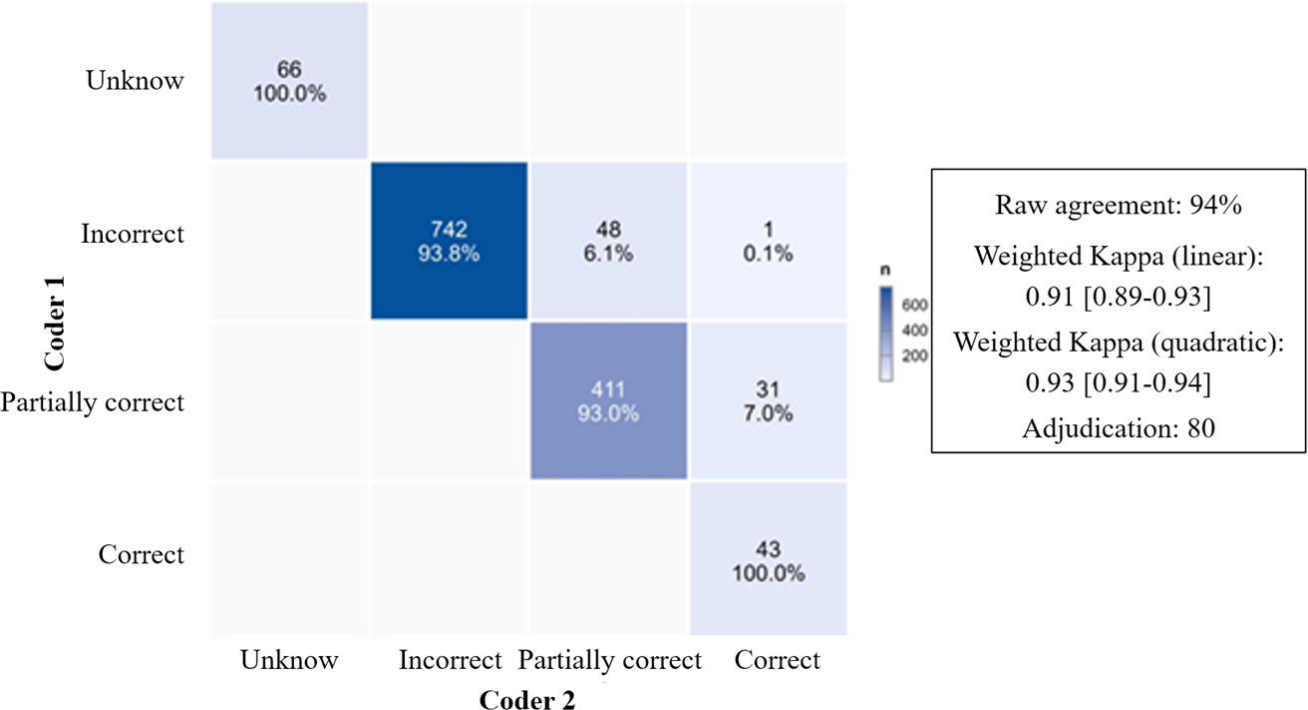
iInterrater agreement for AI definition coding.

The raw agreement between the 2 coders was 94.0% (Figure 2). The weighted Cohen κ was 0.91 (95% CI 0.89‐0.93) using linear weights and 0.93 (95% CI 0.91‐0.94) using quadratic weights (Figure 2). A total of 80 definitions required adjudication (Figure 2). Disagreements were uncommon and mainly involved adjacent categories. Across final AI definition categories, age, academic year, career aspirations, and class ranking did not differ significantly (Table 2). Effect sizes for these comparisons were uniformly small (all Cramér *V*≤0.048; ε²=0.000 for age). Overall, associations between the AI definition category and students’ characteristics, perceptions, and educational expectations were limited in magnitude.

**Table 2.** Characteristics, perceptions, and educational expectations according to final AI definition category^a^.

Variable	Overall (N=1342)	Unknown (n=66)	Incorrect (n=770)	Partially correct (n=439)	Correct (n=67)	*P* value	Effect size
Age, (y), median (IQR)	23 (22-24)	23 (22-24)	23 (22-24)	23 (22-24)	23 (22,23)	.63	ε²=0.000
Academic year, n (%)						.66	Cramér *V*=0.039
Fourth	423 (31.5)	18 (27.3)	240 (31.2)	148 (33.7)	17 (25.4)		
Fifth	674 (50.2)	35 (53.0)	395 (51.3)	210 (47.8)	34 (50.7)		
Sixth	245 (18.3)	13 (19.7)	135 (17.5)	81 (18.5)	16 (23.9)		
Career aspiration, n (%)							
Hospital	775 (57.7)	37 (56.1)	435 (56.5)	264 (60.1)	39 (58.2)	.66	Cramér *V*=0.035
Research	382 (28.5)	19 (28.8)	210 (27.3)	132 (30.1)	21 (31.3)	.71	Cramér *V*=0.032
Private practitioner	502 (37.4)	25 (37.9)	285 (37.0)	167 (38.0)	25 (37.3)	.99	Cramér *V*=0.010
Class ranking, n (%)						.40	Cramér *V*=0.048
Lower third	190 (14.2)	7 (10.6)	117 (15.2)	58 (13.2)	8 (11.9)		
Middle third	694 (51.7)	38 (57.6)	402 (52.2)	225 (51.3)	29 (43.3)		
Upper third	458 (34.1)	21 (31.8)	251 (32.6)	156 (35.5)	30 (44.8)		
General attitude toward AI (0‐10), median (IQR)	7 (5-8)	6 (5-8)	7 (5-8)	7 (6-8)	7 (5.5‐8)	.02	ε²=0.005
Perceived timeline before AI will impact career, n (%)						.18	Cramér *V*=0.070
<5 years	570 (42.5)	28 (42.4)	318 (41.3)	196 (44.6)	28 (41.8)		
In 5 years	357 (26.6)	16 (24.2)	202 (26.2)	123 (28.0)	16 (23.9)		
In 10 years	349 (26.0)	17 (25.8)	214 (27.8)	103 (23.5)	15 (22.4)		
In 20 years	56 (4.2)	3 (4.5)	31 (4.0)	15 (3.4)	7 (10.4)		
In 50 years	4 (0.3)	1 (1.5)	3 (0.4)	0 (0.0)	0 (0.0)		
Not during my lifetime	6 (0.4)	1 (1.5)	2 (0.3)	2 (0.5)	1 (1.5)		
Preferred place for AI learning, n (%)						.10	Cramér *V*=0.063
In curriculum	586 (43.7)	26 (39.4)	319 (41.4)	207 (47.2%)	34 (50.7)		
Outside curriculum	651 (48.5)	36 (54.5)	389 (50.5)	202 (46.0)	24 (35.8)		
Other or free text	105 (7.8)	4 (6.1)	62 (8.1)	30 (6.8)	9 (13.4)		
Top 3 educational objectives, n (%)							
Identify ways in which AI can improve health care quality	949 (70.7)	48 (72.7)	566 (73.5)	292 (66.5)	43 (64.2)	.04	Cramér *V*=0.078
Identify when the technology is appropriate for a given clinical context	916 (68.3)	48 (72.7)	525 (68.2)	295 (67.2)	48 (71.6)	.75	Cramér *V*=0.030
Identify the ethical implications of AI use in clinical settings	897 (66.8)	40 (60.6)	508 (66.0)	300 (68.3)	49 (73.1)	.38	Cramér *V*=0.048

a Values are presented as median (IQR) or n (%). *P* values were derived from Kruskal-Wallis tests for continuous or score variables and chi-square tests for categorical variables. Effect sizes are reported as ε² for Kruskal-Wallis tests and Cramér V for chi-square tests. For career aspirations and educational objectives, multiple responses were allowed; therefore, percentages may exceed 100%.

Figure 2 shows the interrater agreement with a Cohen κ using linear weights of 0.907 (95% CI 0.887‐0.926) and a Cohen κ using quadratic weights of 0.927 (95% CI 0.909‐0.943).

Table 2 shows that age, academic year, career aspirations, and class rankings are similarly distributed across the different AI definition correctness categories. Students with partially correct or correct definitions display a slightly more positive general attitude toward AI compared with other groups. In addition, identifying ways in which AI can improve health care quality is more frequently cited as a priority educational objective, while no major differences are observed regarding the perceived AI impact timeline or the preferred place for AI learning.

#### Attitudes Toward AI

The general attitude toward AI scores differed significantly across AI definition categories (Kruskal-Wallis *P*=.02), although the effect size was very small (ε²=0.005; Table 2). Post hoc Dunn tests with Holm correction showed that students in the unknown group had lower general attitude scores than those in the incorrect group (adjusted *P*=.01), the partially correct group (adjusted *P*=.01), and the correct group (adjusted *P*=.047). No other pairwise differences were observed (Table 2).

The perceived timeline before AI impact did not significantly differ across AI definition categories (*P*=.18; Cramér *V*=0.070). Similarly, the preferred place for AI learning did not significantly differ according to the AI definition category (*P*=.10; Cramér *V*=0.063; Table 2).

Among the 3 leading educational objectives, only the objective related to identifying ways in which AI can improve health care quality differed significantly across groups (*P*=.04), although the effect size remained small (Cramér *V*=0.078; Table 2). The 2 other leading objectives, identifying when AI is appropriate in a given clinical context and identifying the ethical implications of AI use in clinical settings, did not differ significantly across AI definition categories (Table 2).

**Figure 3.**
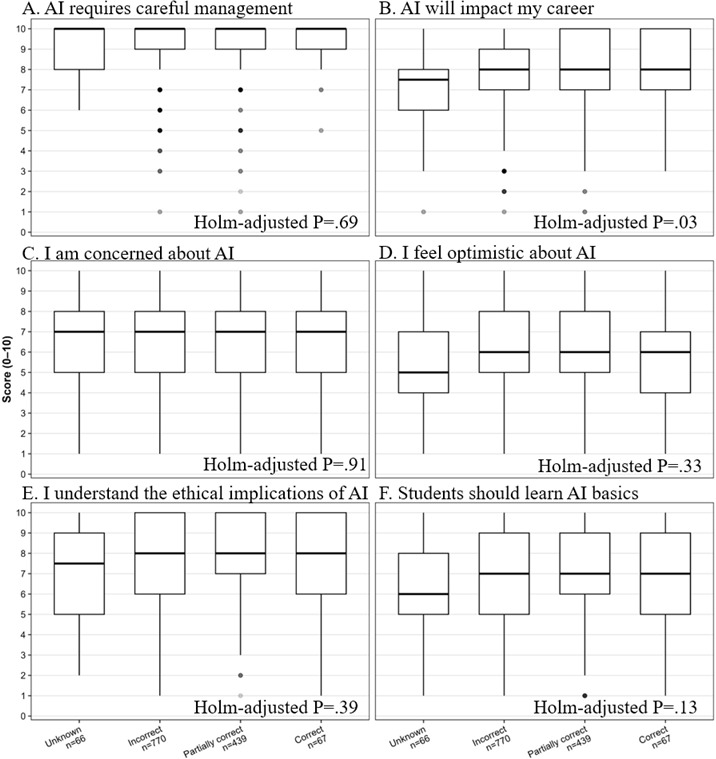
Specific attitudes depending on the result of the AI definition. (A) “AI requires careful management”; (B) “AI will impact my career”; (C) “I am concerned about AI”; (D).”I feel optimistic about AI”; (E) “I understand the ethical implications of AI”; and (F) “Students should learn AI basics.”

Among the 6 specific AI attitude items, the strongest signal concerned the item “AI will impact my career,” which differed across AI definition categories (Kruskal-Wallis *P*=.005; ε²=0.007; Table 2). After Holm correction across the 6 attitude items, this association remained statistically significant (adjusted *P*=.03; Figure 3). Post hoc Dunn-Holm comparisons showed that students in the unknown group reported lower scores than those in the correct group (adjusted *P*=.005) and the partially correct group (adjusted *P*=.02; Table 2 and Figure 3). No other pairwise differences remained significant after correction. The item “students should learn the basics of AI” showed a nominal omnibus association (*P*=.03; ε²=0.005), but this did not remain significant after Holm correction across the 6 items (adjusted *P*=.13; Table 2 and Figure 3). The other 4 specific attitude items were not significantly associated with AI definition category (Table 2 and Figure 3).

Figure 3 presents, via 6 box plots, the distributions of responses (unknown, incorrect, partially correct, and correct) to 5 statements about AI, revealing that the majority of participants assess the impact of AI on their careers with significant differences depending on their response; the other statements do not show any significant differences.

In an exploratory multivariable linear regression model with robust SEs, belonging to the unknown AI definition category remained associated with a lower general attitude toward AI score compared with the incorrect category (regression coefficient β=−0.72, 95% CI −1.17 to −0.28; *P*=.001; Table 3). By contrast, partially correct and correct AI definition categories were not significantly associated with the general attitude score after adjustment. A preference for private practice was independently associated with a higher general attitude toward AI score (regression coefficient β=0.54, 95% CI 0.28-0.79; *P*<.001; Table 3). Older age was also associated with slightly higher scores (regression coefficient β=0.07 per year, 95% CI 0.01-0.12; *P*=.02), whereas being in the sixth year was associated with slightly lower scores compared with the fifth year (regression coefficient β=−0.32, 95% CI −0.61 to −0.03; *P*=.03; Table 3).

**Table 3.** Selected coefficients from the exploratory multivariable linear regression model for the general attitude toward AI score.

Variable	β coefficient, (95% CI)		*P* value
Unknown vs Incorrect AI definition category	−0.72 (−1.17 to −0.28)		.001
Private practice aspiration (yes vs no)	0.54 (0.28 to 0.79)		<.001
Age (y)	0.07 (0.01 to 0.12)		.02
Sixth vs fifth academic year	−0.32 (−0.61 to −0.03)		.03

Table 3 shows the results from an exploratory multivariable linear regression model with heteroskedasticity-consistent robust SEs. The reference categories were the incorrect AI definition category and the fifth academic year. The model was additionally adjusted for the partially correct vs incorrect AI definition category, the correct vs incorrect AI definition category, the fourth vs fifth academic year, class ranking, hospital-based career aspiration, and research aspiration; these associations were not statistically significant after adjustment.

#### Learning

Concerning AI education, 651 (48.5%) preferred AI learning outside the formal curriculum, whereas 586 (43.7%) preferred its inclusion within the curriculum, and 105 (7.8%) responded via free text (Table 1). Regarding the free responses, participants expressed that the training program was too heavy and proposed alternatives such as nonvalidating training, training offered outside the second cycle, or training provided only during internships (Table 4, verbatim 1:4). Finally, some students expressed concerns about the lack of current AI use in clinical settings, which they felt made the training less relevant (Table 4, verbatim 5).

**Table 4.** Verbatim.

Number	Verbatim	Faculty	Age (y)
1	“The knowledge required in medicine is already very extensive; AI training should be non-validating but included in the program.”	Lyon	21
2	“We need the PIX certification to validate our med2med3”	Lille	22
3	“Our learning program is already extremely busy during the externship I struggle to find my place. It should be ““included” in internships. I think. But not necessarily additional courses or training on the side, we already have a lot”	Nancy	23
4	“Should be included later, once AI has a more defined role in”	Lille	23
5	“Should be part of the program when it is truly used on a large scale.”	Lille	24
6	“Intelligence imitating human intelligence with an artificial database.”	Nancy	27
7	“A robot trained to think like a human.”	Lille	22
8	“A search engine that knows almost everything.”	Rennes	24
9	“Computer program that generates information from a set of data and operates on machine learning”	Amiens	23
10	“I love it.”	Lille	25
11	“A major advantage for improving patient care [.]”	Lyon	24
12	“It’s an easily accessible resource that saves time.”	Lille	21
13	“An almost indispensable daily assistant.”	Tours	21
14	“Optimistic. I hope AI will simplify caregivers’ tasks and allow for medicine that is closer to the patient as an individual”	Paris	21
15	“Fear, anger, hatred.”	Marseille	23
16	“Insecurity, it is a tool that can become very dangerous very”	Paris	22
17	“Very mistrustful: outsourcing knowledge dulls our abilities there are security, confidentiality, and ecological concerns.”	Lille	23
18	“I fear we will rely too much on it and become dependent (and stupid in the process AI discourages real thinking)”	Caen	23
19	“Indifference.”	Caen	30
20	“Mixed feelings.”	Nancy	27
21	“AI is the future of just about everything, whether we like it or not. It is up to us to move forward with it and ensure that things are done properly and for the benefit of all.”	Paris	21
22	“A still-imprecise tool that will surely become relevant with more training and supervision by an experienced person.”	Strasbourg	23
23	“A good tool, especially for learning, but it’s important to educate people about its use and the reliability of the information.”	Strasbourg	23
24	“I think AI can be very beneficial as an aid, but its misuse can happen very quickly.”	Lille	22
25	“A lot of potential, but risks of misuse, so it should be introduced with caution.”	Caen	25
26	“Vigilance regarding excessive use.”	Paris	22
27	“A good tool, but dangerous for self-diagnosis, self-medication, and lack of holistic care the human aspect is essential”	Besançon	21
28	“Promising development, but with major challenges due to the type of data it handles (medical information) and possible consequences (patient survival), raising questions of responsibility (the doctor or the machine”	Paris	22
29	“Its proper use requires maintaining a critical mind and understanding the ethical issues behind it.”	Lille	22
30	“Requires ethical and legislative oversight.”	Paris	21
31	“Great potential, but risks changing the profession so much that it may no longer resemble what we are learning now.”	Caen	25
32	“Worrying: the role of the doctor what will be our added value?”	Strasbourg	22
33	“I’m afraid of becoming obsolete.”	Paris	22
34	“Fear of being replaced.”	Lille	22
35	“AI will never replace human contact”	Tours	21
36	“Helps against human error but does not replace the essential.”	Grenoble	39
37	“In medicine, I fear people will trust AI more than their doctor.”	Strasbourg	24
38	“Replacement of support functions previously performed by human professionals loss of professional-patient contact.”	Limoges	31
39	“I think AI is a tool that can help with time-consuming daily tasks (administrative work, data synthesis), but it must be used carefully to avoid dehumanized care.”	Strasbourg	27
40	“Helpful for repetitive tasks.”	Nancy	22
41	“Useful for some courses, but still makes many mistakes interesting for creating flashcards and transferring to Anki afterward.”	Amiens	23
42	“I use AI to structure, condense, and deepen my revisions interactively and personally, especially by creating course.”	Nancy	25
43	“Very helpful for understanding physiopathological concepts in medicine saves time searching through books that often end up contradicting each other.”	Nancy	23
44	“Happy to have access to this resource in my research.”	Lille	24
45	“It helps me understand better, like having a private tutor who questions me, helps me, and analyzes my learning in a targeted”	Nancy	23
46	“This could be a huge step forward in medicine, both diagnostically and administratively, saving a great deal of time, but it must be used wisely as it will never replace humans with their feelings, emotions and consciousness.”	Nancy	23
47	“Can be a valuable aid but should not replace the judgment of a practitioner.”	Amiens	22
48	“I see its value as a diagnostic aid, especially in radiology, but we must never think that AI will replace a doctor.”	Lille	23

Table 1 illustrates opinions on the integration of AI into curricula: 48.5% (n=651) of respondents do not want it included in the curriculum (compared to n=586, 43.7% who do). The preferred formats for learning about AI were short workshops and multiple workshop series, ahead of 1-day courses and graduate-level education. The priority objectives for its introduction are improving health care (25%), understanding how it works (22%), and identifying ethical issues (20%), followed by communication, interpretation of results, and contextual use. This preference for practical and targeted modules reveals a need to combine educational effectiveness and clinical relevance while emphasizing ethics, reflecting a pragmatic and balanced approach between theory, application, and responsibility. The emphasis on workshops suggests a demand for flexibility and gradual immersion.

Table 4 lists the 48 verbatim selections.

Students would mainly prefer training through multiple workshops (Table 1). Additionally, the 3 most frequently selected educational objectives were identifying ways in which AI could improve health care quality (949/1342, 70.7%), identifying when AI is appropriate in a given clinical context (916/1342, 68.3%), and identifying the ethical implications of AI use in clinical settings (897/1342, 66.8%; Table 1). Among the 3 leading educational objectives, only the objective related to identifying ways in which AI could improve health care quality differed significantly across groups (*P*=.04), although the effect size remained small (Cramér *V*=0.078; Table 2). The 2 other leading objectives, identifying when AI is appropriate in a given clinical context and identifying the ethical implications of AI use in clinical settings, did not differ significantly across AI definition categories.

### Qualitative Analysis

#### Coding Reliability

Among the 1342 open-ended responses collected, 1340 were suitable for analysis. Of these, 409 (31%) responses were double-coded independently by 2 researchers to assess coding reliability. An initial disagreement was identified in 18 cases, corresponding to an intercoder agreement rate of 95%. No additional themes emerged during the double-coding process. Following a discussion of the 18 initial disagreements, 9 codes remained unchanged, while 9 codes were reassigned to different themes or split into multiple themes. No further arbitration by a third reviewer was required.

#### Identified Themes

Regarding the analysis of incorrect responses for the AI definition, we identified 3 themes: their representation of AI, AI use, and nuanced optimism (Multimedia Appendix 2). When students expressed their feelings, we again identified the themes of Critical Consideration and Replacement.

#### Their Representation

Students describe AI as a computer tool, software, or a robot, while some compare it to a human. Some perceive AI as a database with notions of infinity or omnipotence; others as an improved search engine, or they directly mention ChatGPT (Table 4, verbatim 6:9).

#### Nuanced Optimism

Students express positive aspects, described as help that can be indispensable, effective, and provided daily. According to the students, AI would save time through rapid execution and easy access. Additionally, some explain that the use of AI will improve patient care and provide an opportunity to focus more on the relationship with the patient (Table 4, verbatim 10:14). In contrast, some share feelings of anger and fear, highlighting dangers, particularly regarding energy costs, the dehumanization of medicine, and the regression of skills (Table 4, verbatim 15:18). Finally, some students express neutral, doubtful, uncertain, and sometimes fatalistic views, stating that AI is part of the future (Table 4, verbatim 19; 20).

#### Critical Consideration

Students expressed some mistrust toward AI, which is fallible and imperfect, requiring learning and human control (Table 4, verbatim 21; 22). Additionally, students expressed reservations about how AI is used, particularly regarding excessive use or restrictions for patients (Table 4, verbatim 23:26). Finally, students questioned data security issues, the need for a precise framework for use with established ethical rules, and information about responsibility in case of error (Table 4, verbatim 27:29).

#### Replacement

With the advancement of AI in health care, students questioned the role of doctors and the AI-related changes in their work (Table 4, verbatim 30). Some described a feeling of fear, while others believed replacement by AI was impossible due to the need for doctor-patient relationships and empathy (Table 4, verbatim 31:34). There was also concern about patients using AI, which could lead to a loss of credibility and a weakening of the doctor–patient relationship (Table 4, verbatim 35:37).

#### AI Use

Students describe varied uses, such as assistance with administrative tasks and data management (Table 4, verbatim 38; 39). Additionally, some express benefits for their learning, including understanding lessons, creating study sheets, receiving personalized training, and searching for information (Table 4, verbatim 40:44). Finally, regarding clinical applications, students express divergent views on whether AI should be used for clinical reasoning and decision-making support, with particularly polarized opinions in the field of radiology (Table 4, verbatim 45:48).

## Discussion

### Principal Results

This study aimed to explore medical students’ representations and attitudes toward AI in France in 2025. Our results indicate that students’ attitudes toward AI depend essentially on their knowledge of the definition of AI, as students with more accurate definitions tend to report more positive attitudes. There is also great variability in students’ attitudes toward AI, with marked differences not only between participants but also within individual responses. These differences highlight the complexity of student perceptions of AI. The themes of “nuanced optimism” and “replacement” align with observations from other studies [18,23,24,26]. New themes also emerged, such as fear of skill loss, awareness of the high ecological cost of AI use, and loss of credibility with patients. Concerns about skill loss have also been reported among educators, particularly regarding the risk of cognitive offloading and erosion of critical thinking skills, suggesting that this apprehension is shared across different stakeholders in medical education [10,11]. Ecological concerns, meanwhile, align with broader societal discussions on the energy consumption and environmental impact of large-scale AI systems, transcending the specific context of medical education [36].

Additionally, the results reveal how AI is used for learning and searching for information on the internet. However, the need for an ethical framework and responsible AI use remains a major concern for students, even though the World Health Organization (WHO) recommendations have been in place since 2021 [2].

Our study reveals 2 notable developments compared to previous work. First, only a small percentage of students reported not knowing how to define AI, compared to half of the participants in the studies by Teng et al [21]. In contrast, in our study, half of the students gave incorrect answers, suggesting that students now develop representations of AI, even if they are often erroneous, rather than being completely unaware. Their concerns about ethical considerations, AI management, and its impact on their careers remain similar to those observed by Teng et al [21]. As noted by Amiri et al [19], students’ prior knowledge strongly influences their perceptions. However, neither the level of education nor the academic year showed a significant effect [19]. These results are likely related to self-assessment of their ranking within their class. Finally, the theme of “replacement” persists [19,21], as does ambivalence between perceived AI utility and its risks. Finally, unlike in some studies [18,23,26], no formal training request emerged, probably due to students’ self-training or the still-limited clinical use of AI.

We also highlighted a form of critical consideration among students toward AI. Indeed, students expressed ethical concerns and reservations about using AI in medical contexts [20]. For example, they emphasized the need for a precise framework and established ethical rules, but this critical attitude seems less present when it comes to their own learning. Students show some mistrust toward AI as a clinical tool, recognizing its limitations and the need for human supervision. However, this critical consideration is less evident in their use of AI for learning, where they seem more inclined to accept the provided information without always questioning it. This dichotomy suggests that students perceive greater risks related to AI in medical practice than in their own learning process. It would, therefore, be crucial to encourage a more critical approach to AI use in learning, emphasizing the importance of verifying and validating the obtained information to develop a more thoughtful and responsible use of these technological tools [37].

AI use by medical students is still relatively unexplored compared with studies in other educational fields [30,32,38]. Our study revealed that medical students use AI in various ways to meet their learning needs, primarily for writing assistance, information searches, or personalized learning. It seems that this use in their learning could lead to excessive and uncritical use of information. Moreover, it is crucial to explore the impact on skill acquisition and dependence on AI in their professional environments. However, this adoption of AI in their learning process could potentially result in excessive and uncritical use of the provided information, risking the creation of dependence on these tools. On the other hand, this could also lead to the loss of essential skills, particularly regarding clinical judgment and decision-making autonomy, which are crucial in medical practice. Therefore, it is essential to explore these potential impacts on both the acquisition of critical medical skills and students’ future professional practice.

### Limitations

Our study had certain limitations. First, the response rate relative to the number of enrolled students was less than 10%, which limits the generalizability of our findings. The use of voluntary participation may have introduced self-selection bias, as students with stronger preexisting interest in AI, whether positive or negative, may have been more likely to respond. While participation exceeded 10% at some universities, the low overall rate suggests that our results may disproportionately reflect the attitudes of a more engaged subgroup rather than the broader student population. Second, we chose the Teng et al [21] survey because it was designed 4 years ago, allowing for comparison. Its mixed methodology offered the advantage of exploring both the underlying reasons for students’ attitudes and their modes of AI use [20]. However, regarding the evaluation of the definition, it is important to note that it is not possible to assess students’ knowledge based solely on their ability to define AI. Other surveys that evaluate AI knowledge more globally could have been used, but their length might have discouraged participation and thus reduced the response rate. A key limitation of our study is the absence of demographic data, including gender or sexual orientation. We deliberately chose not to collect this information. This decision was motivated by two ethical concerns: (1) the need to avoid reductive or exclusionary categorizations of gender, which is a spectrum of identities extending beyond binary frameworks; and (2) the risk of compromising participant anonymity, as detailed demographic data could have enabled indirect identification in smaller faculty samples. Finally, although several associations reached statistical significance due to the large sample size, the magnitudes are uniformly small, and practical implications should be interpreted cautiously.

### Conclusions

Our study describes the current perceptions, attitudes, and uses of AI among French medical students in 2025. Students report generally positive attitudes toward AI despite limited conceptual knowledge of the definition of AI, alongside concerns related to ethics, ecological impact, and potential skill loss. Many already use AI tools for learning in a largely self-directed manner. Further longitudinal research is needed to better understand the implications of these practices for medical education and professional development. These findings provide a descriptive baseline for future evaluations of AI-related training and its impact on learning and professional development.

## Supplementary material

10.2196/91345 Multimedia Appendix 1 Full survey.

10.2196/91345 Multimedia Appendix 2 Mind Map qualitative results.

10.2196/91345 Checklist 1 COREQ checklist.

## Abbreviations

COREQ: Consolidated Criteria for Reporting Qualitative Research
GDPR: General Data Protection Regulation
OECD: Organisation for Economic Co-operation and Development
SAQ: short open-ended question
WHO: World Health Organization
