# Supplementary material for: Perceptions, Attitudes, and Use of AI by Medical Students: Mixed Methods Study
Source: JMIR Med Educ. 2026 Jul 20;12:e91345. doi: 10.2196/91345 (PMC13384426; doi:10.2196/91345)
Supplement: Multimedia Appendix 1 [file mededu-v12-e91345-s001.pdf]

Full Survey

1. Describe Artificial Intelligence (AI) in one sentence. Please enter "Don't know" if applicable

____

2. On a scale of 0 to 10, how much do you support or oppose AI development in your field of study

1	2	3	4	5	6	7	8	9	10

strongly oppose strongly support

On a scale of 0 to 10, how much do you support or oppose AI development in your field of study:

0 = strongly oppose 5 = neutral 10 = strongly support

3. I believe artificial intelligence will have an impact on my career.

1	2	3	4	5	6	7	8	9	10

disagree completely agree completely

4. I believe healthcare students need to learn the basics of AI.

1	2	3	4	5	6	7	8	9	10

disagree completely agree completely

5. I understand the ethical implications of AI usage in my field.

1	2	3	4	5	6	7	8	9	10

disagree completely agree completely

6. I feel hopeful about having AI in my field.

1 2 3 4 5 6 7 8 9 10

disagree completely agree completely

7. I am worried about the role AI will play in my field

1 2 3 4 5 6 7 8 9 10

disagree completely agree completely

8. I believe AI is a technology that requires careful management.

1 2 3 4 5 6 7 8 9 10

disagree completely agree completely

9. Please use one word or sentence to describe how you feel about AI in your field:

____

10. If your program were to introduce AI basics, which 3 objectives would be most important to you? Drag and drop here

- Identify when technology is appropriate for a given clinical context
- Understand and interpret AI-generated results
- Be able to communicate how the technology works in a way that others can understand
- Identify the ethical implications of using AI in clinical contexts
- Understand how the underlying technological processes work
- Learn the terminologies in order to communicate and collaborate with engineers/developers
- Identify ways AI can improve healthcare quality improvement
- Other:

11. À How soon do you think AI will impact your career?

- 5 years
- in 10 years
- in 20 years
- in 50 years
- not in my lifetime

12. Should learning about AI basics be part of your curriculum, or should it be outside of curriculum time (extracurricular)?

- Should be a part of my program/curriculum
- Should be outside of curriculum time
- Other

13. Which of the following would you be interested in attending to learn more about AI basics? (can select multiple)

- 1-day course
- Multiple workshop series
- 1- or 2-hour workshop
- Graduate-level education (Master's, PhD)
- Other

14. How old are you? (in years)

____

15. Which medical school are you attending?

____

16. In general, which of the following statements apply to you?

- I want to pursue research in my future career
- I want to start my own practice/business in the future
- I want to focus solely on clinical work

17. Which medical speciality or specialities interest you? *

- Anatomical Pathology
- Anaesthesia
- Cardiology
- Cardiac Surgery
- Digestive Surgery
- Orthopaedic Surgery
- Plastic Surgery
- Paediatric Surgery
- Dermatology
- Endocrinology
- Gastroenterology
- Genetics
- Geriatrics
- Gynaecology/Obstetrics
- General Medicine
- Internal medicine
- Occupational medicine
- Emergency medicine
- Nephrology
- Nuclear medicine
- Neurosurgery
- Neurology
- Oncology
- Ophthalmology
- Otorhinolaryngology (ENT)
- Paediatrics
- Pulmonology
- Psychiatry
- Radiology
- Intensive care
- Rheumatology
- Public health
- Urology
- None

18. Where do you rank in your medical school class?

- I rank in the top third
- I rank in the middle third
- I rank in the bottom third

19. (optional) Thank you so much for participating in our survey! If you'd like to enter the draw to win a $20 gift card to Amazon or Tim Hortons, please enter your email address below.
