# Supplementary material for: Perceptions, Attitudes, and Use of AI by Medical Students: Mixed Methods Study
Source: JMIR Med Educ. 2026 Jul 20;12:e91345. doi: 10.2196/91345 (PMC13384426; doi:10.2196/91345)
Supplement: Checklist 1 [file mededu-v12-e91345-s003.pdf]

Domain	Item Number	COREQ Item	Response
Domain 1: Research team and reflexivity	1	Interviewer/facilitator: Which author conducted the interview?	Data collection was designed and administered online by Frédéric Paris. The survey and manuscript were reviewed by his supervisor, Laure Abensur Vuillaume.
Domain 1: Research team and reflexivity	2	Credentials: What were the researcher's credentials?	Frédéric Paris holds a Master's degree. Laure Abensur Vuillaume holds a PhD.
Domain 1: Research team and reflexivity	3	Occupation: What was their occupation at the time of the study?	At the time of the study, Frédéric Paris worked part-time in clinical practice and part-time in research, while completing a Master's degree in Health Sciences Education.
Domain 1: Research team and reflexivity	4	Gender: Was the researcher male or female?	Frédéric Paris is male; Laure Abensur Vuillaume is female.
Domain 1: Research team and reflexivity	5	Experience and training: What experience or training did the researcher have?	Frédéric Paris had no prior experience in qualitative research. Laure Abensur Vuillaume had authored approximately 30
Domain 1: Relationship with participants	6	Relationship established: Was a relationship established prior to study commencement?	No prior relationship was established with participants, as the survey was anonymous and conducted online.
Domain 1: Relationship with participants	7	Participant knowledge of the interviewer: What did participants know about the researcher?	Participants knew that the purpose of the study was to explore their perceptions and feelings regarding artificial intelligence (AI).
Domain 1: Relationship with participants	8	Interviewer characteristics: Any characteristics reported about the interviewer?	Individuals with either strong positive or strong negative views about AI may have been more motivated to participate, potentially influencing the nature of responses.
Domain 2: Study design	9	Methodological orientation: Grounded theory,	A thematic analysis approach was used.
Domain 2: Study design	10	Sampling: How were participants selected?	Snowball sampling was employed.
Domain 2: Study design	11	Method of approach: In person, mail, phone, etc.?	Participants were recruited through email invitations and social media dissemination.
Domain 2: Study design	12	Sample size: How many participants were in the study?	A total of 1,342 participants completed the survey.
Domain 2: Study design	13	Non-participation: Reasons for non-participation?	Thirty-five participants were excluded, primarily because they did not meet the inclusion criteria.

Domain 2: Setting	14	Setting of data collection: Where was the data collected?	Data were collected online, allowing participants to respond from any setting of their choice.
Domain 2: Setting	15	Presence of non-participants: Anyone else present during	Unknown, as responses were collected through an online form.
Domain 2: Setting	16	Description of sample: Important characteristics of participants?	The sample had a mean age of 23.1 years (IQR 22–24), and most were in their 5th year of study. Most participants (57,7%) planned a clinical career, and 52% were in the middle third of their class academically.
Domain 2: Data collection	17	Interview guide: Were questions or prompts provided?	A structured online survey was used.
Domain 2: Data collection	18	Repeat interviews: Were repeat interviews carried out?	The questionnaire was pilot-tested with six medical students. They were organized in three pairs and instructed to verbalize confusion and report issues. Two errors were identified and corrected: a duplicated question and a multiple-choice item incorrectly limited to a single response. The revised version was then completed without issues.
Domain 2: Data collection	19	Audio/visual recording: Was recording used?	No recordings were made.
Domain 2: Data collection	20	Field notes: Were field notes made?	No field notes were taken.
Domain 2: Data collection	21	Duration: What was the duration of interviews?	The survey required approximately five minutes to complete.
Domain 2: Data collection	22	Data saturation: Was saturation discussed?	Data saturation was not discussed.
Domain 2: Data collection	23	Transcripts returned: Were transcripts returned to participants?	No transcripts or responses were returned to participants for validation.
Domain 3: Analysis and findings	24	Number of data coders: How many coders were involved?	One primary coder analyzed the data. A second coder reviewed 30% of the dataset to ensure consistency.
Domain 3: Analysis and findings	25	Description of coding tree: Was a coding tree described?	No coding tree was provided.

Domain 3: Analysis and findings	26	Derivation of themes: How were themes derived?	Themes were derived using a mixed approach. Some themes (e.g., “nuanced optimism,” “replacement concerns”) had been identified in previous literature, while additional themes were developed deductively from the data.
Domain 3: Analysis and findings	27	Software: What software was used?	Microsoft Excel was used for data management and analysis.
Domain 3: Analysis and findings	28	Participant checking: Did participants provide feedback?	Participants did not review the findings.
Domain 3: Reporting	29	Quotations presented: Were participant quotations used?	Illustrative participant quotes were included, accompanied by age and university city for context.
Domain 3: Reporting	30	Data and findings consistent: Was there consistency?	Yes, there was consistency between the raw data and the findings.
Domain 3: Reporting	31	Clarity of major themes: Were major themes clearly presented?	Major themes were clearly presented.
Domain 3: Reporting	32	Clarity of minor themes: Minor or diverse cases described?	Minor and divergent themes were also described.
